# Supplementary material for: Use of autobiographical stimuli as a mood manipulation procedure: Systematic mapping review
Source: PLoS One. 2022 Jun 27;17(6):e0269381. doi: 10.1371/journal.pone.0269381 (PMC9236260; doi:10.1371/journal.pone.0269381)
Supplement: S1 File — (DOCX) [file pone.0269381.s002.docx]

**S1: Checklist: Template for a Mapping Study Protocol**

| **Section Recommendation** | **Line #** |
| --- | --- |
| 1. **Change Record** |  |
| This should be a list or table summarizing the main updates and changes embodied in each version of the protocol and (where appropriate), the reasons for these | **N/A** |
| 1. **Background** |  |
| 1. Explain why there is a need for a study on this topic 2. Identify the topic that to be “scoped” in the study 3. Specify any research questions that will be addressed 4. If extending previous research on the topic, explain why a new study is needed | **53-65**  **65-69**  **87-101**  **N/A** |
| 1. **Search Strategy** |  |
| 1. Specify and justify basic strategy: manual search, automated search, or mixed. 2. For automated searches, specify search terms and compounds of these (and record results of any prototyping of the search strings) 3. For automated searches, identify resources to be used (digital libraries and search engines) 4. For manual searches, identify the journals and conferences to be searched 5. Specify the time period to be covered by the review and any reasons for your choice 6. Identify any ancillary search procedures, e.g. asking leading researchers or research groups, or accessing their web sites; or checking reference lists of primary studies 7. Specify how the search process is to be evaluated (e.g. against a known subset of papers; or against the results from a previous systematic review or mapping study) | **N/A**  **106-112**  **106-112**  **N/A**  **120-123**  **128-129**  **N/A** |
| 1. **Selection Criteria** 2. identify the inclusion criteria for primary studies 3. identify the exclusion criteria 4. define how selection will be undertaken (roles of analysts) 5. define how agreement among analysts will be evaluated 6. define how any differences between analysts will be resolved | **116-125**  **126-133**  **136-138**  **N/A**  **148-154** |
| 1. **Data Extraction** 2. Design data extraction form (and check via a dry run) 3. Specify the strategy for extracting the data and the form (paper, on-line etc.) 4. Identify how the data extraction process is to be undertaken and validated, particularly any data that require numerical calculations, or are subjective | **N/A** |
| 1. **Synthesis** 2. Specify the categorization schemes to be used 3. Assess the threats to validity (construct, internal, external), particularly constraints on the search process and deviations from standard practice | **627-640** |
| 1. **Study Limitations**   Specify residual validity issues including potential conflicts of interest (i.e. that are inherent in the context of the study, rather than arising from the plan). | **627-640** |
| 1. **Reporting**   Identify target audience, relationship to other studies, planned publications, authors of the publications. | **N/A** |
| 1. **Schedule**   Provide time estimates for all of the major steps. | **N/A** |
