# Supplementary material for: Use of autobiographical stimuli as a mood manipulation procedure: Systematic mapping review
Source: PLoS One. 2022 Jun 27;17(6):e0269381. doi: 10.1371/journal.pone.0269381 (PMC9236260; doi:10.1371/journal.pone.0269381)
Supplement: S2 File — (DOCX) [file pone.0269381.s003.docx]

**S2: Bibliography of the studies included in the review**

P1: Albarracin D, Hart W. Positive Mood + Action = Negative Mood + Inaction: Effects of General Action and Inaction Concepts on Decisions and Performance as a Function of Affect. *Emotion*. 2011;11(4):951–7. doi: 10.1037/a0024130

P2: Albarracin D, Kumkale T. Affect as information in persuasion: A model of affect identification and discounting. *J Pers Soc Psychol*. 2003;84(3):453–69. doi: [10.1037/0022-3514.84.3.453](https://psycnet.apa.org/doi/10.1037/0022-3514.84.3.453)

P3: Albarracín D, Wyer RS. Elaborative and nonelaborative processing of a behavior-related communication. *Personal Soc Psychol Bull*. 2001;27(6):691–705. doi: 10.1177/0146167201276005

P4: Allen RJ, Schaefer A, Falcon T. Recollecting positive and negative autobiographical memories disrupts working memory. *Acta Psychol*. 2014;151:237–43. doi: 10.1016/j.actpsy.2014.07.003

P5: Arshamian A, Iannilli E, Gerber JC, Willander J, Persson J, Seo HS, et al. The functional neuroanatomy of odor evoked autobiographical memories cued by odors and words. *Neuropsychologia*. 2013;51(1):123–31. doi: 10.1016/j.neuropsychologia.2012.10.023

P6: Baldwin M, Biemat M, Landau MJ. Remembering the real me: Nostalgia offers a window to the intrinsic self. *J Pers Soc Psychol*. 2015;108(1):128–47. doi: 10.1037/a0038033

P7: Barliya A, Omlor L, Giese MA, Berthoz A, Flash T. Expression of emotion in the kinematics of locomotion. *Exp Brain Res*. 2013;225(2):159–76. doi: 10.1007/s00221-012-3357-4

P8: Barrett FS, Grimm KJ, Robins RW, Wildschut T, Sedikides C, Janata P. Music-Evoked Nostalgia: Affect, Memory, and Personality. *Emotion*. 2010;10(3):390–403. doi: 10.1037/a0019006

P9: Barrett FS, Janata P. Neural responses to nostalgia-evoking music modeled by elements of dynamic musical structure and individual differences in affective traits. *Neuropsychologia*. 2016;91:234–46. doi: 10.1016/j.neuropsychologia.2016.08.012

P10: Baumann J, DeSteno D. Emotion Guided Threat Detection: Expecting Guns Where There Are None. *J Pers Soc Psychol*. 2010;99(4):595–610. doi: 10.1037/a0020665

P11: Becker MW, Leinenger M. Attentional selection is biased toward mood-congruent stimuli. *Emotion*. 2011;11(5):1248–54. doi: 10.1037/a0023524

P12: Benuzzi F, Ballotta D, Handjaras G, Leo A, Papale P, Zucchelli M, et al. Eight weddings and six funerals: An fMRI study on autobiographical memories. *Front Behav Neurosci*. 2018;12:1–11. doi: 10.3389/fnbech.2018.00212

P13: Bluck S, Alea N. Characteristics of positive autobiographical memories in adulthood. *Int J Aging Hum Dev*. 2009;69(4):247–65. doi:10.2190/AG.69.4.a

P14: Boyacioglu I, Akfirat S, Yılmaz AE. Gender differences in emotional experiences across childhood, romantic relationship, and self-defining memories. *J Cogn Psychol*. 2017;29(2):137–50. doi: 10.1080/20445911.2016.1216996

P15: Briñol P, Petty RE, Barden J. Happiness Versus Sadness as a Determinant of Thought Confidence in Persuasion: A Self-Validation Analysis. *J Pers Soc Psychol*. 2007;93(5):711–27. doi: 10.1037/0022-3514.93.5.711

P16: Burns JW, Kubilus A, Bruehl S. Emotion induction moderates effects of anger management style on acute pain sensitivity. *Pain*. 2003;106(1–2):109–18. doi: 10.1016/S0304-3959(03)00298-7

P17: Cady ET, Harris RJ, Knappenberger JB. Using music to cue autobiographical memories of different lifetime periods. *Psychol Music*. 2008;36(2):157–77. doi: 10.1177/0305735607085010

P18: Carretero LM, Latorre JM, Fernández D, Barry TJ, Ricarte JJ. Effects of positive personal and non-personal autobiographical stimuli on emotional regulation in older adults. *Aging Clin Exp Res*. 2020;32(1):157–64. doi: 10.1007/s40520-019-01147-0

P19: Cerqueira CT, Almeida JRC, Gorenstein C, Gentil V, Leite CC, Sato JR, et al. Engagement of multifocal neural circuits during recall of autobiographical happy events. *Brazilian J Med Biol Res*. 2008;41(12):1076–85. doi: [10.1590/s0100-879x2008001200006](https://doi.org/10.1590/s0100-879x2008001200006)

P20: Cerqueira CT, Almeida JRC, Sato JR, Gorenstein C, Gentil V, Leite CC, et al. Cognitive control associated with irritability induction: An autobiographical recall fMRI study. *Rev Bras Psiquiatr*. 2010 Jun;32(2):109–18. doi: 10.1590/s1516-44462010000200004

P21: Chu S, Downes JJ. Proust nose best: Odors are better cues of autobiographical memory. *Mem Cogn*. 2002;30(4):511–8. doi:10.3758/bf03194952

P22: Clark IA, Mackay CE, Holmes EA. Positive involuntary autobiographical memories: You first have to live them. *Conscious Cogn*. 2013;22(2):402–6. doi: 10.1016/j.concog.2013.01.008

P23: Cohen AS, Lee Hong S, Guevara A. Understanding emotional expression using prosodic analysis of natural speech: Refining the methodology. *J Behav Ther Exp Psychiatry*. 2010;41(2):150–7. doi:10.1016/j.jbtep.2009.11.008

P24: Cooney RE, Joormann J, Atlas LY, Eugène F, Gotlib IH. Remembering the good times: Neural correlates of affect regulation. *Neuroreport*. 2007;18(17):1771–4.

P25: Damasio AR, Grabowski TJ, Bechara A, Damasio H, Ponto LLB, Parvizi J, et al. Subcortical and cortical brain activity during the feeling of self-generated emotions. *Nat Neurosci*. 2000 Oct;3(10):1049–56. doi:10.1038/79871

P26: Dasgupta N, DeSteno D, Williams LA, Hunsinger M. Fanning the Flames of Prejudice: The Influence of Specific Incidental Emotions on Implicit Prejudice. *Emotion*. 2009;9(4):585–91. doi: 10.1037/a0015961

P27: Debeer E, Raes F, Williams JMG, Craeynest M, Hermans D. Operant conditioning of autobiographical memory retrieval. *Memory*. 2014;22(3):171–83. doi: 10.1080/09658211.2013.774419

P28: de Bruijn MJ, Bender M. Olfactory cues are more effective than visual cues in experimentally triggering autobiographical memories. *Memory*. 2018;26(4):547–58. doi:10.1080/09658211.2017.1381744

P29: Dehghani A, Soltanian-Zadeh H, Hossein-Zadeh GA. Global Data-Driven Analysis of Brain Connectivity during Emotion Regulation by Electroencephalography Neurofeedback. *Brain Connect*. 2020;10(6):302–15. doi: 10.1089/brain.2019.0734

P30: Demorest AP. Anger, compassion, and happiness as antidotes for sadness. *Am J Psychol*. 2019;132(2):227–36. doi:10.5406/amerjpsyc.132.2.0227

P31: Denkova E, Botzung A, Scheiber C, Manning L. Implicit emotion during recollection of past events: A nonverbal fMRI study. *Brain Res*. 2006;1078(1):143–50. doi: 10.1016/j.brainres.2006.01.061

P32: Denkova E, Dolcos S, Dolcos F. The effect of retrieval focus and emotional valence on the medial temporal lobe activity during autobiographical recollection. *Front Behav Neurosci*. 2013;7:109. doi: 10.3389/fnbeh.2013.00109

P33: Denkova E, Dolcos S, Dolcos F. Neural correlates of “distracting” from emotion during autobiographical recollection. *Soc Cogn Affect Neurosci*. 2015;10(2):219–30. doi:10.1093/scan/nsu039

P34: Escobedo JR, Adolphs R. Becoming a better person: Temporal remoteness biases autobiographical memories for moral events. *Emotion*. 2010;10(4):511–8. doi: 10.1037/a0018723

P35: Fabiansson EC, Denson TF, Moulds ML, Grisham JR, Schira MM. Don’t look back in anger: Neural correlates of reappraisal, analytical rumination, and angry rumination during recall of an anger-inducing autobiographical memory. *Neuroimage*. 2012;59(3):2974–81. doi:10.1016/j.neuroimage.2011.09.078

P36: Fawver B, Hass CJ, Park KD, Janelle CM. Autobiographically recalled emotional states impact forward gait initiation as a function of motivational direction. *Emotion*. 2014;14(6):1125–36. doi:10.1037/a0037597

P37: Fishbach A, Labroo AA. Be Better or Be Merry: How Mood Affects Self-Control. *J Pers Soc Psychol*. 2007;93(2):158–73. doi:10.1037/0022-3514.93.2.158

P38: Ford JH, Kensinger EA. The role of the amygdala in emotional experience during retrieval of personal memories. *Memory*. 2019;27(10):1362–70. doi: 10.1080/09658211.2019.1659371

P39: Ford JH, Rubin DC, Giovanello KS. Effects of task instruction on autobiographical memory specificity in young and older adults. *Memory*. 2014;22(6):722–36. doi:10.1080/09658211.2013.820325

P40: Gadeikis D, Bos N, Schweizer S, Murphy F, Dunn B. Engaging in an experiential processing mode increases positive emotional response during recall of pleasant autobiographical memories. *Behav Res Ther*. 2017;92:68–76. doi:10.1016/j.brat.2017.02.005

P41: Gendolla GHE, Abele AE, Krüsken J. The Informational Impact of Mood on Effort Mobilization: A Study of Cardiovascular and Electrodermal Responses. *Emotion*. 2001;1(1):12–24. doi:10.1037/1528-3542.1.1.12

P42: Gendolla GHE, Krüsken J. Mood state, task demand, and effort-related cardiovascular response. *Cogn Emot*. 2002;16(5):577–603. doi: 10.1080/02699930143000446

P43: Gillihan SJ, Kessler J, Farah MJ. Memories affect mood: Evidence from covert experimental assignment to positive, neutral, and negative memory recall. *Acta Psychol*. 2007;125(2):144–54. doi:10.1016/j.actpsy.2006.07.009

P44: Goetz MC, Goetz PW, Robinson MD. What’s the Use of Being Happy? Mood States, Useful Objects, and Repetition Priming Effects. *Emotion*. 2007;7(3):675–9. doi:10.1037/1528-3542.7.3.675

P45: Goldin PR, Moodie CA, Gross JJ. Acceptance versus reappraisal: Behavioral, autonomic, and neural effects. *Cogn Affect Behav Neurosci*. 2019;19(4):927–44. doi:10.3758/s13415-019-00690-7

P46: Göritz AS, Moser K. Web-based mood induction. *Cogn Emot*. 2006;20(6):887–96. doi: 10.1080/02699930500405386

P47: Griskevicius V, Shiota MN, Neufeld SL. Influence of Different Positive Emotions on Persuasion Processing: A Functional Evolutionary Approach. *Emotion*. 2010;10(2):190–206. doi:10.1037/a0018421

P48: Gross MM, Crane EA, Fredrickson BL. Methodology for Assessing Bodily Expression of Emotion. *J Nonverbal Behav*. 2010;34(4):223–48. doi: 10.1007/s10919-010-0094-x

P49: Hernandez S, Vander Wal JS, Spring B. A negative mood induction procedure with efficacy across repeated administrations in women. *J Psychopathol Behav Assess*. 2003;25(1):49–55. doi:10.1023/A:1022252020196

P50: Herz RS, Eliassen J, Beland S, Souza T. Neuroimaging evidence for the emotional potency of odor-evoked memory. *Neuropsychologia*. 2004;42(3):371–8. doi:10.1016/j.neuropsychologia.2003.08.009

P51: Herz RS, Schooler JW. A naturalistic study of autobiographical memories evoked by olfactory and visual cues: Testing the Proustian hypothesis. *Am J Psychol*. 2002;115(1):21–32.

P52: Houle I, Philippe FL. Need satisfaction in episodic memories impacts mood at retrieval and well-being over time. *Pers Individ Dif*. 2017;105:194–9. doi:10.1016/j.paid.2016.09.059

P53: Iordan AD, Dolcos S, Dolcos F. Brain Activity and Network Interactions in the Impact of Internal Emotional Distraction. *Cereb Cortex*. 2019;29(6):2607–23. doi:10.1093/cercor/bhy129

P54: Jacques PLS, Conway MA, Cabeza R. Gender differences in autobiographical memory for everyday events: Retrieval elicited by SenseCam images versus verbal cues. *Memory*. 2011;19(7):723–32. doi:10.1080/09658211.2010.516266

P55: Jahanitabesh A, Cardwell BA, Halberstadt J. Sadness and ruminative thinking independently depress people’s moods. *Int J Psychol*. 2019;54(3):360–8. doi: 10.1002/ijop.12466

P56: Jakubowski K, Belfi AM, Eerola T. Phenomenological differences in music - and television -evoked autobiographical memories. *Music Percept.* 2021;38(5):435–55. doi:10.1525/mp.2021.38.5.435

P57: Jakubowski K, Ghosh A. Music-evoked autobiographical memories in everyday life. *Psychol Music*. 2021;49(3):649–66. doi:10.1177/0305735619888803

P58: Jallais C, Gilet AL. Inducing changes in arousal and valence: Comparison of two mood induction procedures. *Behav Res Methods*. 2010;42(1):318–25. doi: 10.3758/BRM.42.1.318

P59: Janata P. The neural architecture of music-evoked autobiographical memories. *Cereb Cortex*. 2009;19(11):2579–94. doi:10.1093/cercor/bhp008

P60: Janata P, Tomic ST, Rakowski SK. Characterisation of music-evoked autobiographical memories. *Memory*. 2007;15(8):845–60. doi: 10.1080/09658210701734593

P61: Jefferies LN, Smilek D, Eich E, Enns JT. Emotional valence and arousal interact in attentional control: Research article. *Psychol Sci*. 2008;19(3):290–5. doi: 10.1111/j.1467-9280.2008.02082

P62: Jeon YA, Resnik SN, Feder GI, Kim K. Effects of emotion-induced self-focused attention on item and source memory. *Motiv Emot*. 2020;44(5):719–37. doi: 10.1007/s11031-020-09830-w

P63: Kemps E, Tiggemann M. Reducing the vividness and emotional impact of distressing autobiographical memories: The importance of modality-specific interference. *Memory*. 2007;15(4):412–22. doi:10.1080/09658210701262017

P64: Kenworthy JB, Canales CJ, Weaver KD, Miller N. Negative incidental affect and mood congruency in crossed categorization. *J Exp Soc Psychol*. 2003;39(3):195–219. doi:10.1016/S0022-1031(03)00022-2

P65: Kneeland ET, Nolen-Hoeksema S, Dovidio JF, Gruber J. Beliefs about emotion’s malleability influence state emotion regulation. *Motiv Emot*. 2016;40(5):740–9. doi:10.1007/s11031-016-9566-6

P66: Kohn N, Falkenberg I, Kellermann T, Eickhoff SB, Gur RC, Habel U. Neural correlates of effective and ineffective mood induction. *Soc Cogn Affect Neurosci*. 2013;9(6):864–72. doi:10.1093/scan/nst055

P67: Krackow E, Kania K, Travers RM. Does Negative Mood Confer an Advantage in the Recall of Negative Life Events? *Imagin Cogn Pers*. 2013;32(3):291–305. doi: [10.2190/IC.32.3.e](https://doi.org/10.2190/IC.32.3.e)

P68: Kristen-Antonow S. The role of ToM in creating a reminiscence bump for MEAMs from adolescence. *Psychol Music*. 2019;47(1):51–68. doi: 10.1177/0305735617735374

P69: Kross E, Davidson M, Weber J, Ochsner K. Coping with Emotions Past: The Neural Bases of Regulating Affect Associated with Negative Autobiographical Memories. *Biol Psychiatry*. 2009;65(5):361–6. doi: 10.1016/j.biopsych.2008.10.019

P70: Laco M, Polatsek P, Dekrét Š, Benesova W, Baránková M, Strnádelová B, et al. Effects of individual’s emotions on saliency and visual search. *Vis Comput*. 2020;37:1581-1592. doi:10.1007/s00371-020-01912-7

P71: Lane RD, McRae K, Reiman EM, Chen K, Ahern GL, Thayer JF. Neural correlates of heart rate variability during emotion. *Neuroimage*. 2009;44(1):213–22. doi:10.1016/j.neuroimage.2008.07.056

P72: Lench HC, Levine LJ. Effects of fear on risk and control judgements and memory: Implications for health promotion messages. *Cogn Emot*. 2005;19(7):1049–69. doi:10.1080/02699930500203112

P73: Lemer JS, Keltner D. Fear , Anger , and Risk. 2001;81(1):146–59. doi: [10.1037//0022-3514.81.1.146](https://doi.org/10.1037//0022-3514.81.1.146)

P74: Lievaart M, Huijding J, van der Veen FM, Hovens JE, Franken IHA. The impact of angry rumination on anger-primed cognitive control. *J Behav Ther Exp Psychiatry*. 2017;54:135–42. doi:10.1016/j.jbtep.2016.07.016

P75: Liotti M, Mayberg HS, Brannan SK, McGinnis S, Jerabek P, Fox PT. Differential limbic-cortical correlates of sadness and anxiety in healthy subjects: Implications for affective disorders. *Biol Psychiatry*. 2000;48(1):30–42. doi:10.1016/s0006-3223(00)00874-x

P76: Lobbestael J, Arntz A, Wiers RW. How to push someone’s buttons: A comparison of four anger-induction methods. *Cogn Emot*. 2008;22(2):353–73. doi:10.1080/02699930701438285

P77: López-Cano MA, Navarro B, Nieto M, Andrés-Pretel F, Latorre JM. Autobiographical emotional induction in older people through popular songs: Effect of reminiscence bump and enculturation. *PLoS One*. 2020;15(9). doi: 10.1371/journal.pone.0238434

P78: MacKinnon S, Gevirtz R, McCraty R, Brown M. Utilizing heartbeat evoked potentials to identify cardiac regulation of vagal afferents during emotion and resonant breathing. *Appl Psychophysiol Biofeedback*. 2013;38(4):241–55. doi: 10.1007/s10484-013-9226-5

P79: Maki Y, Janssen SMJ, Uemiya A, Naka M. The phenomenology and temporal distributions of autobiographical memories elicited with emotional and neutral cue words. *Memory*. 2013;21(3):286–300. doi:10.1080/09658211.2012.725739

P80: Markowitsch HJ, Vandekerckhove MMP, Lanfermann H, Russ MO. Engagement of lateral and medial prefrontal areas in the ecphory of sad and happy autobiographical memories. *Cortex*. 2003;39(4–5):643–65. doi:10.1016/s0010-9452(08)70858-x

P81: Masaoka Y, Sugiyama H, Katayama A, Kashiwagi M, Homma I. Slow breathing and emotions associated with odor-induced autobiographical memories. *Chem Senses*. 2012;37(4):379–88. doi:10.1093/chemse/bjr120

P82: Matsunaga M, Bai Y, Yamakawa K, Toyama A, Kashiwagi M, Fukuda K, et al. Brain-Immune Interaction Accompanying Odor-Evoked Autobiographic Memory. *PLoS One*. 2013;8(8):1–9. doi:10.1371/journal.pone.0072523

P83: Matsunaga M, Isowa T, Yamakawa K, Kawanishi Y, Tsuboi H, Kaneko H, et al. Psychological and physiological responses to odor-evoked autobiographic memory. *Neuroendocrinol Lett*. 2011;32(6):774–80.

P84: Mills C, D’Mello S. On the validity of the autobiographical emotional memory task for emotion induction. *PLoS One*. 2014;9(4). doi: 10.1371/journal.pone.0095837

P85: Molins F, Pérez-Calleja T, Abad-Tortosa D, Alacreu-Crespo A, Serrano-Rosa MÁ. Positive emotion induction improves cardiovascular coping with a cognitive task. *PeerJ*. 2021;12. doi:10.7717/peerj.10904:

P86: Nawa NE, Ando H. Effective connectivity within the ventromedial prefrontal cortex-hippocampus-amygdala network during the elaboration of emotional autobiographical memories. *Neuroimage*. 2019;189:316–28. doi: 10.1016/j.neuroimage.2019.01.042

P87: Oba K, Noriuchi M, Atomi T, Moriguchi Y, Kikuchi Y. Memory and reward systems coproduce “nostalgic” experiences in the brain. *Soc Cogn Affect Neurosci*. 2016;11(7):1069–77. doi:10.1093/scan/nsv073

P88: Öner S, Gülgöz S. Autobiographical remembering regulates emotions: a functional perspective. *Memory*. 2018;26(1):15–28. doi: 10.1080/09658211.2017.1316510

P89: Ozawa S. Emotions Induced by Recalling Memories About Interpersonal Stress. *Front Psychol*. 2021;12. doi:10.3389/fpsyg.2021.618676

P90: Pacheco-Unguetti AP, Parmentier FBR. Sadness increases distraction by auditory deviant stimuli. *Emotion*. 2014;14(1):203–13. doi:10.1037/a0034289

P91: Pacheco-Unguetti AP, Parmentier FBR. Happiness increases distraction by auditory deviant stimuli. *Br J Psychol*. 2016;107(3):419–33. doi: 10.1111/bjop.12148

P92: Pelletier M, Bouthillier A, Lévesque J, Carrier S, Breault C, Paquette V, et al. Separate neural circuits for primary emotions? Brain activity during self-induced sadness and happiness in professional actors. *Neuroreport*. 2003;14(8):1111–6. doi:10.1097/00001756-200306110-00003

P93: Perreau-Linck E, Beauregard M, Gravel P, Paquette V, Soucy JP, Diksic M, et al. In vivo measurements of brain trapping of 11C-labelled α-methyl-L-tryptophan during acute changes in mood states. *J Psychiatry Neurosci*. 2007;32(6):430–4.

P94: Philippot P, Schaefer A, Herbette G. Consequences of specific processing of emotional information: Impact of general versus specific autobiographical memory priming on emotion elicitation. *Emotion*. 2003;3(3):270–83. doi: 10.1037/1528-3542.3.3.270

P95: Platz F, Kopiez R, Hasselhorn J, Wolf A. The impact of song-specific age and affective qualities of popular songs on music-evoked autobiographical memories (MEAMs). *Music Sci*. 2015;19(4):327–49. doi:10.1177/1029864915597567

P96: Pólya T. Temporal structure of narratives reveals the intensity of the narrator’s current affective state. *Curr Psychol*. 2021;40(1):281–91. doi:10.1007/s12144-018-9921-8

P97: Rainville P, Bechara A, Naqvi N, Damasio AR. Basic emotions are associated with distinct patterns of cardiorespiratory activity. *Int J Psychophysiol*. 2006;61(1):5–18. doi:10.1016/j.ijpsycho.2005.10.024

P98: Ramírez E, Ortega AR, Chamorro A, Colmenero JM. A program of positive intervention in the elderly: Memories, gratitude and forgiveness. *Aging Ment Heal*. 2014;18(4):463–70. doi:10.1080/13607863.2013.856858

P99: Razumnikova O, Khoroshavtseva E. Imbalance between emotionally negative and positive life events retrieval and the associated asymmetry of brain activity. *Behav Sci*. 2020;10(1). doi:10.3390/bs10010018

P100: Reid CA, Green JD, Wildschut T, Sedikides C. Scent-evoked nostalgia. *Memory*. 2015;23(2):157–66. doi:10.1080/09658211.2013.876048

P101: Richter M, Gendolla GHE. Mood impact on cardiovascular reactivity when task difficulty is unclear. *Motiv Emot*. 2009;33(3):239–48. doi:10.1007/s11031-009-9134-4

P102: Riener CR, Stefanucci JK, Proffitt DR, Clore G. An effect of mood on the perception of geographical slant. *Cogn Emot*. 2011;25(1):174–82. doi: 10.1080/026999310033738026

P103: Roisman GI, Fortuna K, Holland A. An experimental manipulation of retrospectively defined earned and continuous attachment security. *Child Dev*. 2006;77(1):59–71. doi:10.1111/j.1467-8624.2006.00856.x

P104: Sagliano L, Trojano L, Di Mauro V, Carnevale P, Di Domenico M, Cozzolino C, et al. Attentional biases for threat after fear-related autobiographical recall. *Anxiety, Stress Coping*. 2018;31(1):69–78. doi:10.1080/10615806.2017.1362297

P105: Seebauer L, Arthen T, Austermann M, Falck J, Koch L, Moulds ML, et al. Mood repair in healthy individuals: Both processing mode and imagery content matter. *J Behav Ther Exp Psychiatry*. 2016;50:289–94. doi:10.1016/j.jbtep.2015.10.005

P106: Selcuk E, Zayas V, Günaydin G, Hazan C, Kross E. Mental representations of attachment figures facilitate recovery following upsetting autobiographical memory recall. *J Pers Soc Psychol*. 2012;103(2):362–78. doi:10.1037/a0028125

P107: Sheldon S, Donahue J. More than a feeling: Emotional cues impact the access and experience of autobiographical memories. *Mem Cogn*. 2017;45(5):731–44. doi:10.3758/s13421-017-0691-6

P108: Siedlecka E, Capper MM, Denson TF. Negative emotional events that people ruminate about feel closer in time. *PLoS One*. 2015;10(2):1–18. doi: 10.1371/journal.pone.0117105

P109: Sitaram R, Lee S, Ruiz S, Rana M, Veit R, Birbaumer N. Real-time support vector classification and feedback of multiple emotional brain states. *Neuroimage*. 2011;56(2):753–65. doi:10.1016/j.neuroimage.2010.08.007

P110: Sugimori E, Shimokawa K, Aoyama Y, Kita T, Kusumi T. Empathetic listening boosts nostalgia levels and positive emotions in autobiographical narrators. *Heliyon*. 2020;6(8). doi:10.1016/j.heliyon.2020.e04546

P111: Tang D, Schmeichel BJ. Stopping anger and anxiety: Evidence that inhibitory ability predicts negative emotional responding. *Cogn Emot*. 2014;28(1):132–42. doi:10.1080/02699931.2013.799459

P112: Trilla I, Weigand A, Dziobek I. Affective states influence emotion perception: evidence for emotional egocentricity. *Psychol Res*. 2021;85(3):1005–15. doi:10.1007/s00426-020-01314-3

P113: Tsai MH, Young MJ. Anger, fear, and escalation of commitment. *Cogn Emot*. 2010;24(6):962–73. doi:10.1080/02699930903050631

P114: Vanderlind WM, Stanton CH, Weinbrecht A, Velkoff EA, Joormann J. Remembering the Good Ole Days: Fear of Positive Emotion Relates to Affect Repair Using Positive Memories. *Cognit Ther Res*. 2017;41(3):362–8. doi: 10.1007/s10608-016-9775-z

P115: van Schie CC, Chiu C De, Rombouts SARB, Heiser WJ, Elzinga BM. When I relive a positive me: Vivid autobiographical memories facilitate autonoetic brain activation and enhance mood. *Hum Brain Mapp*. 2019;40(16):4859–71. doi: 10.1002/hbm.24742

P116: Vuoskoski JK, Eerola T. Can sad music really make you sad? indirect measures of affective states induced by music and autobiographical memories. *Psychol Aesthetics, Creat Arts*. 2012;6(3):204–13. doi:10.1037/a0026937

P117: Watanabe K, Masaoka Y, Kawamura M, Yoshida M, Koiwa N, Yoshikawa A, et al. Left Posterior Orbitofrontal Cortex Is Associated With Odor-Induced Autobiographical Memory: An fMRI Study. *Front Psychol*. 2018. 9:687. doi: 10.3389/fpysg.2018.00687

P118: Willander J, Larsson M. Olfaction and emotion: The case of autobiographical memory. *Mem Cogn*. 2007;35(7):1659–63. doi:10.3758/bf03193499

P119: Willander J, Larsson M. The mind’s nose and autobiographical odor memory. *Chemosens Percept*. 2008;1(3):210–5. doi:10.1007/s12078-008-9026-0

P120: Willander J, Sikström S, Karlsson K. Multimodal retrieval of autobiographical memories: Sensory information contributes differently to the recollection of events. *Front Psychol*. 2015;6:1681. doi:10.3389/fpysg.2015.01681

P121: Wolf T, Demiray B. The mood-enhancement function of autobiographical memories: Comparisons with other functions in terms of emotional valence. *Conscious Cogn*. 2019;70:88–100. doi:10.1016/j.concog.2019.03.002

P122: Young KD. Differential effects of emotionally versus neutrally cued autobiographical memories on performance of a subsequent cognitive task: effects of task difficulty. *Front Psychol*. 2012;3:299. doi: 10.3389/fpsyg.2012.00299

P123: Young MJ, Tiedens LZ, Jung H, Tsai MH. Mad enough to see the other side: Anger and the search for disconfirming information. *Cogn Emot*. 2011;25(1):10–21. doi:10.1080/02699930903534105

P124: Zator K, Katz AN. The language used in describing autobiographical memories prompted by life period visually presented verbal cues, event-specific visually presented verbal cues and short musical clips of popular music. *Memory*. 2017;25(6):831–44. doi:10.1080/09658211.2016.1224353

P125: Zhang X, Yu HW, Barrett LF. How does this make you feel? A comparison of four affect induction procedures. *Front Psychol*. 2014;5:689. doi: 10.3389/fpsyg.2014.00689

P126: Zotev V, Krueger F, Phillips R, Alvarez RP, Simmons WK, Bellgowan P, et al. Self-regulation of amygdala activation using real-time FMRI neurofeedback. *PLoS One*. 2011;6(9). doi:10.1371/journal.pone.0024522
