# Supplementary material for: Use of autobiographical stimuli as a mood manipulation procedure: Systematic mapping review
Source: PLoS One. 2022 Jun 27;17(6):e0269381. doi: 10.1371/journal.pone.0269381 (PMC9236260; doi:10.1371/journal.pone.0269381)
Supplement: S3 File — (DOCX) [file pone.0269381.s004.docx]

| **ID** | **Author (Year)** | **Country*** | **Journal** | **Area** | **Cue type** | **Emotional state under study** |
| --- | --- | --- | --- | --- | --- | --- |
| P1 | Albarracin & Hart (2011) | United States of America | Emotion | Experimental psychology | Verbal | Happyness, anger |
| P2 | Albarracin & Kumkale (2003) | United States of America | Journal of Personality and Social Psychology | Social psychology | Verbal | Happyness, anger |
| P3 | Albarracin & Wyer (2001) | United States of America | Personality and Social Psychology Bulletin | Social psychology | Verbal | Happyness, anger |
| P4 | Allen et al. (2014) | United Kingdom | Acta Psychologica | Experimental psychology | Verbal | Positive, negative |
| P5 | Arshamian et al. (2013) | Germany | Neuropsychologia | Behavior sciences | Verbal and olfactory | Positives |
| P6 | Baldwin et al. (2015) | United States of America | Journal of Personality and Social Psychology | Social psychology | Verbal | Nostalgia |
| P7 | Barliya et al. (2013) | Israel | Experimental Brain Research | Neuroscience | Verbal | Anger, fear, sadness, joy |
| P8 | Barrett et al. (2010) | United States of America | Emotion | Experimental psychology | Musical | Positives, negatives |
| P9 | Barrett & Janata (2016) | United States of America | Neuropsychologia | Behavior sciences | Musical | Happiness, sadness |
| P10 | Baumann & DeSteno (2010) | United States of America | Journal of Personality and Social Psychology | Social psychology | Verbal | Anger, happiness |
| P11 | Becker & Leinenger (2011) | United States of America | Emotion | Experimental psychology | Verbal | Happiness, sadness |
| P12 | Benuzzi et al. (2018) | Italy | Frontiers in Behavioral Neuroscience | Behavior sciences | Verbal | Positives, negatives |
| P13 | Bluck & Alea (2009) | United States of America | The International Journal of Aging and Human Development | Development psychology | Verbal | Positives |
| P14 | Boyacioglu et al. (2016) | Turkey | Journal of Cognitive Psychology | Experimental psychology | Verbal | Positives, negatives |
| P15 | Briñol et al. (2007) | Spain | Journal of Personality and Social Psychology | Social psychology | Verbal | Happiness, sadness |
| P16 | Burns et al. (2003) | United States of America | Pain | Medicine and experimental research | Verbal | Fear, sadness, joy |
| P17 | Cady et al. (2008) | United States of America | Psychology of Music | Experimental psychology | Musical and visual | Positives, negatives |
| P18 | Carretero et al. (2020) | Spain | Aging Clinical and Experimental Research | Geriatric and gerontology | Visual | Positives, negatives |
| P19 | Cerqueira et al. (2008) | Brazil | Brazilian Journal of Medical and Biological Research | Medicine and experimental research | Verbal | Happiness, irritability |
| P20 | Cerqueira et al. (2010) | Brazil | Revista Brasileira de Psiquiatría | Psychiatry | Verbal | Happiness, irritability |
| P21 | Chu & Downes (2002) | United Kingdom | Motivation and Emotion | Experimental psychology | Verbal and olfactory | N/D |
| P22 | Clark et al. (2013) | United Kingdom | Consciousness and Cognition | Experimental psychology | Visual | Positives |
| P23 | Cohen et al. (2010) | United States of America | Journal of Behavior Therapy and Experimental Psychiatry | Clinical psychology | Verbal | N/D |
| P24 | Cooney et al. (2007) | United States of America | NeuroReport | Neurosciences | Verbal | Positives, sadness |
| P25 | Damasio et al. (2000) | United States of America | Nature Neuroscience | Neurosciences | Verbal | Happiness, sadness, fear, anger |
| P26 | Dasgupta et al. (2009) | United States of America | Emotion | Experimental psychology | Verbal and visual | Anger, disgust |
| P27 | Debeer et al. (2013) | Belgium | Memory | Experimental psychology | Verbal | Positives, negatives |
| P28 | de Brujin & Bender (2017) | The Netherlands | Memory | Experimental psychology | Olfactory | Positives, negatives |
| P29 | Dehghani et al. (2020) | Iran | Brain Connectivity | Neurosciences | Verbal | Positives |
| P30 | Demorest (2019) | United States of America | The American Journal of Psychology | Multidisciplinary psychology | Verbal | Happiness, sadness, anger |
| P31 | Denkova et al. (2006) | France | Brain Research | Neurosciences | Visual | N/D |
| P32 | Denkova et al. (2013) | France | Frontiers in Behavioral Neuroscience | Behavior sciences | Verbal | Positives, negatives |
| P33 | Denkova et al. (2015) | France | Social Cognitive and Affective Neuroscience | Neurosciences | Verbal | Positives, negatives |
| P34 | Escobedo & Adolphs (2010) | United States of America | Emotion | Experimental psychology | Verbal | Positives, negatives |
| P35 | Fabiansson et al. (2012) | Australia | NeuroImage | Neurosciences | Verbal | Anger |
| P36 | Fawver et al. (2014) | United States of America | Emotion | Experimental psychology | Verbal | Anger, fear, happiness, sadness |
| P37 | Fishbach & Labroo (2007) | United States of America | Journal of Personality and Social Psychology | Social psychology | Verbal | Happiness, unhappiness |
| P38 | Ford & Kensinger (2019) | United States of America | Memory | Experimental psychology | Visual | Positives, negatives |
| P39 | Ford et al. (2014) | United States of America | Memory | Experimental psychology | Musical | N/D |
| P40 | Gadeikis et al. (2017) | United Kingdom | Behaviour Research and Therapy | Experimental psychology | Verbal | Happiness |
| P41 | Gendolla et al. (2001) | Germany | Emotion | Experimental psychology | Verbal | Positives, negatives |
| P42 | Gendolla & Krüsken (2002) | Germany | Cognition and Emotion | Experimental psychology | Verbal | Positives, negatives |
| P43 | Gillihan et al. (2007) | United States of America | Acta Psychologica | Experimental psychology | Verbal | Positives, negatives |
| P44 | Goetz et al. (2007) | United States of America | Emotion | Experimental psychology | Verbal | Happiness, sadness |
| P45 | Goldin et al. (2019) | United States of America | Cognitive, Affective, and Behavioral Neuroscience | Behavior sciences | Verbal | N/D |
| P46 | Görtiz & Moser (2006) | Germany | Cognition and Emotion | Experimental psychology | Verbal | Positives, negatives |
| P47 | Griskevicius et al. (2010) | United States of America | Emotion | Experimental psychology | Verbal | N/D |
| P48 | Gross et al. (2010) | United States of America | Journal of Nonverbal Behavior | Social psychology | Verbal | Positives, negatives |
| P49 | Hernández et al. (2003) | United States of America | Journal of Psychopathology and Behavioral Assessment | Clinical psychology | Musical | Negatives |
| P50 | Herz et al. (2004) | United States of America | Neuropsychologia | Behavior sciences | Olfactory and visual | Positives, negatives |
| P51 | Herz & Schooler (2002) | United States of America | The American Journal of Psychology | Multidisciplinary psychology | Olfactory, verbal, and visual | N/D |
| P52 | Houle & Philippe (2017) | Canada | Personality and Individual Differences | Social psychology | Verbal | Positives, negatives |
| P53 | Iordan et al. (2019) | United States of America | Cerebral Cortex | Neurosciences | Verbal | Negatives |
| P54 | Jacques et al. (2011) | United States of America | Memory | Experimental psychology | Verbal and visual | Negatives |
| P55 | Jahanitabesh et al. (2017) | Iran | International Journal of Psychology | Multidisciplinary psychology | Verbal and musical | Sadness |
| P56 | Jakubowski et al. (2021) | United Kingdom | Music Perception | Experimental psychology | Verbal | N/D |
| P57 | Jakubowski & Ghosh (2019) | United Kingdom | Psychology of Music | Experimental psychology | Musical | Positives mixed |
| P58 | Jallais & Gilet (2010) | France | Behavior Research Methods | Experimental psychology | Verbal and musical | Happiness, serenity, anger, sadness |
| P59 | Janata (2009) | United States of America | Cerebral Cortex | Neurosciences | Musical | Positives, negatives |
| P60 | Janata et al. (2007) | United States of America | Memory | Experimental psychology | Musical | Positives, negatives |
| P61 | Jefferies et al. (2008) | Canada | Psychological Science | Multidisciplinary psychology | Verbal and musical | Anxiety, sadness, happiness, calm |
| P62 | Jeon et al. (2020) | United States of America | Motivation and Emotion | Experimental psychology | Verbal | Shame, sadness |
| P63 | Kemps & Tiggemann (2007) | Australia | Memory | Experimental psychology | Verbal | Happiness, distressing |
| P64 | Kenworthy et al. (2003) | United States of America | Journal of Experimental Social Psychology | Social psychology | Verbal | Sadness, anger |
| P65 | Kneedland et al. (2016) | United States of America | Motivation and Emotion | Experimental psychology | Verbal | Negatives |
| P66 | Kohn et al. (2014) | Germany | Social Cognitive and Affective Neuroscience | Neurosciences | Verbal and visual | Happiness |
| P67 | Krackow et al. (2013) | United States of America | Imagination, Cognition and Personality | Experimental psychology | Verbal | Positives, negatives |
| P68 | Kristen-Antonow (2019) | Germany | Psychology of Music | Experimental psychology | Musical | N/D |
| P69 | Kross et al. (2009) | United States of America | Biological Psychiatry | Neurosciences | Verbal | Negatives |
| P70 | Laco et al. (2021) | Slovak Republic | The Visual Computer | Computer graphics | Verbal | Happiness |
| P71 | Lane et al. (2009) | United States of America | NeuroImage | Neurosciences | Verbal | Sadness |
| P72 | Lerner & Keltner (2001) | United States of America | Journal of Personality and Social Psychology | Social psychology | Verbal | Anger, fear |
| P73 | Lench & Levine (2005) | United States of America | Cognition and Emotion | Experimental psychology | Verbal | Fear, anger, happiness |
| P74 | Lievaart et al. (2017) | The Netherlands | Journal of Behavior Therapy and Experimental Psychiatry | Clinical psychology | Verbal | Anger |
| P75 | Liotti et al. (2000) | United States of America | Society of Biological Psychiatry | Neurosciences | Verbal | Anxiety, sadness |
| P76 | Lobbestael et al. (2008) | The Netherlands | Cognition and Emotion | Experimental psychology | Verbal | Anger, positives |
| P77 | López-Cano et al. (2020) | Spain | Plos One | Multidisciplinary psychology | Musical | N/D |
| P78 | MacKinnon et al. (2013) | United States of America | Applied Psychophysiology and Biofeedback | Clinical psychology | Verbal | Happiness, sadness |
| P79 | Maki et al. (2013) | Japan | Memory | Experimental psychology | Verbal | N/D |
| P80 | Markowitsch et al. (2003) | Germany | Cortex | Neurosciences | Verbal | Happiness, sadness |
| P81 | Masaoka et al. (2012) | Japan | Chemical Senses | Neurosciences | Olfactory | Pleasant, unpleasant |
| P82 | Matsunaga et al. (2013) | Japan | Plos One | Multidisciplinary psychology | Olfactory | Nostalgia |
| P83 | Matsunaga et al. (2011) | Japan | Neuro Endocrinology Letters | Neurosciences | Olfactory | Nostalgia |
| P84 | Mills & D´Mello (2014) | United States of America | Plos One | Multidisciplinary psychology | Verbal | Anger, fear |
| P85 | Molins et al. (2021) | Spain | Peer J | Multidisciplinary psychology | Verbal | N/D |
| P86 | Nawa & Ando (2019) | Japan | NeuroImage | Neurosciences | Verbal | Positives, negatives |
| P87 | Oba et al. (2016) | Japan | Social Cognitive and Affective Neuroscience | Neurosciences | Visual | Nostalgia |
| P88 | Öner & Gúlgóz (2017) | Turkey | Memory | Experimental psychology | Verbal | Sadness, anger |
| P89 | Ozawa (2021) | Japan | Frontiers in Psychology | Multidisciplinary psychology | Verbal | Stress |
| P90 | Pacheco-Unguetti & Parmentier (2014) | Spain | Emotion | Experimental psychology | Musical | Sadness |
| P91 | Pacheco-Unguetti & Parmentier (2016) | Spain | British Journal of Psychology | Multidisciplinary psychology | Musical | Happiness |
| P92 | Pelletier et al. (2003) | Canada | NeuroReport | Neurosciences | Verbal | Happiness, sadness |
| P93 | Perreau-Linck et al. (2007) | Canada | Journal of Psychiatry and Neuroscience | Neurosciences | Verbal | Happiness, sadness |
| P94 | Philippot et al. (2003) | Belgium | Emotion | Experimental psychology | Verbal | N/D |
| P95 | Platz et al. (2015) | Germany | Musicae Scientiae | Experimental psychology | Musical | N/D |
| P96 | Pólya (2021) | Hungary | Current Psychology | Multidisciplinary psychology | Verbal | Anger, sadness, joy, pride |
| P97 | Rainville et al. (2006) | Canada | International Journal of Psychophysiology | Neurosciences | Verbal | Happiness, fear |
| P98 | Ramirez et al. (2014) | Spain | Aging and Mental Health | Geriatric and gerontology | Musical and visual | Positives |
| P99 | Razumnikova & Khoroshavtseva (2020) | Russia | Behavioral Sciences | Behavior sciences | Verbal | Happiness, joy, anger, sadness |
| P100 | Reid et al. (2015) | United States of America | Memory | Experimental psychology | Olfactory | Nostalgia |
| P101 | Richter & Gendolla (2009) | Switzerland | Motivation and Emotion | Experimental psychology | Verbal | Happiness, sadness |
| P102 | Riener et al. (2011) | United States of America | Cognition and Emotion | Experimental psychology | Verbal | Positives, negatives |
| P103 | Roisman et al. (2006) | United States of America | Child Development | Development psychology | Verbal and musical | Happiness, sadness |
| P104 | Sagliano et al. (2018) | Italy | Anxiety, Stress, and Coping | Multidisciplinary psychology | Verbal | Happiness, fear |
| P105 | Seebauer et al. (2016) | Germany | Journal of Behavior Therapy and Experimental Psychiatry | Clinical psychology | Verbal | Positives |
| P106 | Selcuk et al. (2012) | United States of America | Journal of Personality and Social Psychology | Social psychology | Verbal | Negatives |
| P107 | Sheldon & Donahue (2017) | Canada | Memory and Cognition | Experimental psychology | Musical | Happiness, fear, sadness, paceful |
| P108 | Siedlecka et al. (2015) | Australia | Plos One | Multidisciplinary psychology | Verbal | Anger |
| P109 | Sitaram et al. (2011) | Germany | NeuroImage | Neurosciences | Verbal | Happiness, disgust, sadness |
| P110 | Sugimori et al. (2020) | Japan | Heliyon | Multidisciplinary psychology | Musical | Nostalgia |
| P111 | Tang et al. (2014) | United States of America | Cognition and Emotion | Experimental psychology | Musical | Anger, anxiety |
| P112 | Trilla et al. (2021) | Germany | Psychological Research | Social psychology | Verbal | Happiness, sadness |
| P113 | Tsai et al. (2010) | United States of America | Cognition and Emotion | Experimental psychology | Verbal | Anger, fear |
| P114 | Vanderlind et al. (2017) | United States of America | Cognitive Therapy and Research | Clinical psychology | Verbal | Positives |
| P115 | vanSchie et al. (2019) | The Netherlands | Human Brain Mapping | Neurosciences | Verbal | Positives |
| P116 | Vuoskoski & Eerola (2012) | Finland | Psychology of Aesthetics, Creativity, and the Arts | Experimental psychology | Musical | Anger, fear, sadness |
| P117 | Watanabe et al. (2018) | Japan | Frontiers in Psychology | Multidisciplinary psychology | Olfactory | N/D |
| P118 | Willander & Larsson (2007) | Sweden | Memory and Cognition | Experimental psychology | Verbal and olfactory | N/D |
| P119 | Willander & Larsson (2008) | Sweden | Chemosensory Perception | Neurosciences | Olfactory | N/D |
| P120 | Willander et al. (2015) | Sweden | Frontiers in Psychology | Multidisciplinary psychology | Musical, olfactory, and visual | N/D |
| P121 | Wolf & Demiray (2019) | Germany | Consciousness and Cognition | Experimental psychology | Verbal | Positives, negatives |
| P122 | Young et al. (2012) | United States of America | Frontiers in Psychology | Multidisciplinary psychology | Verbal | Anger |
| P123 | Young et al. (2011) | United States of America | Cognition and Emotion | Experimental psychology | Verbal | Positives, negatives |
| P124 | Zator & Katz (2017) | Canada | Memory | Experimental psychology | Verbal and musical | N/D |
| P125 | Zhang et al. (2014) | United States of America | Frontiers in Psychology | Multidisciplinary psychology | Verbal and musical | Anger, joy |
| P126 | Zotev et al. (2011) | United States of America | Plos One | Multidisciplinary psychology | Verbal | Happiness |

*Notes*: * = according to the affiliation of the first author.; N/D: Not Definied
