# Supplementary material for: Use of autobiographical stimuli as a mood manipulation procedure: Systematic mapping review
Source: PLoS One. 2022 Jun 27;17(6):e0269381. doi: 10.1371/journal.pone.0269381 (PMC9236260; doi:10.1371/journal.pone.0269381)
Supplement: Attachment — Submitted filename: Response letter to reviewers Mapping-2-2.docx [file pone.0269381.s005.docx]

**Response letter to reviewers.**

**Ms. Ref. No.: PONE-D-21-39680**

Original Title: Use of autobiographical stimuli as a mood manipulation procedure: Systematic Mapping Review

PLOS ONE

Dear editor and reviewers,

First of all, I would like to thank you and the reviewers once again for your time and dedication in reviewing our article and for your good feedback and positive comments that have been taken into account to improve this article. We agree with all their comments and we have revised our manuscript accordingly.

Thanks to these comments it has been possible to improve the article and I will now go on to detail the changes made to the article. To facilitate the work of the reviewers, in some instances we refer to the former manuscript indicating the page and the line (page-line). We hope that the changes we have made are acceptable but of course we would be willing to work further on the manuscript where necessary.

With kindest regards,

**Reviewers' comments:**

**Reviewer #1:**

We appreciate that the reviewer´s comments. The following are our point-by-point responses:

Please, include script file. This information will help readers to understand the process of your study.

REPLY: The R script will be available to all those interested in it, and this is indicating in the manuscript (p. 10, line 303).

Please, add this reference:
Jordan, Zoe PhD; Lockwood, Craig PhD; Munn, Zachary PhD; Aromataris, Edoardo PhD The updated Joanna Briggs Institute Model of Evidence-Based Healthcare, International Journal of Evidence-Based Healthcare: March 2019 - Volume 17 - Issue 1 - p 58-71 doi: 10.1097/XEB.0000000000000155

REPLY: As suggested by the reviewer, the reference is added to the article (number 22)

please improve tool description

REPLY: In response to the reviewer's request, a brief explanation of the tool used to measure the quality of the included studies is added (p. 7, lines 234-235).

*“This tool of proven validity allows us to analyse the included articles by determining their quality according to the type of design.”*

Pubmed is not a database. PubMed is a free search engine accessing primarily the MEDLINE database

REPLY: In response to the reviewer's request, we add (MEDLINE).

Could you add a wordcloud (or tag cloud)?

REPLY: The word clouds can be found in the document "Figures". Here you can find the word clouds according to journal, authors and grouping by word topics.

**Reviewer #2:**

This manuscript addresses an interesting and important issue in psychological research: Use of autobiographical stimuli as a mood manipulation procedure. The literature can benefit from studies that clarify the mood induction procedures.

We appreciate that the reviewer’s comments. The following are our point-by-point responses:

My comments are organized according to the sections of the paper, with the most important issues in each section addressed first. The following are some concerns and recommendations for strengthening this paper.

Abstract: It would be useful to include a broad description of the conclusions in the abstract.

REPLY We agree with the reviewer and we have included more information about the conclusions in the abstract (p. 2, lines 45-53).

Keywords. “Autobiographical” keyword is redundant with the title. Perhaps, authors could include another keyword

REPLY: As suggested by the reviewer, we have changed “Autobiographical” by “personal memories”

Introduction
The introduction should include some aspect that describes why interest in the study of use of autobiographical stimuli as a mood manipulation procedure has increased (or the benefits of its increase) and what gaps this review intends to cover.

REPLY: We agree with the reviewer that we need to emphasize the interest in the study of autobiographical stimulus as a mood induction procedure. The nature of this mapping review is to find existing gaps throughout the scientific literature to make them known to future researchers. We have now included an explication about this (p. 4, lines 113-125).

Materials & Methods

Authors follow a systematic mapping review approach. It would be rigorous to include some reference that justifies its suitability for the objectives set forth in this work.

REPLY: Thank you for this observation. We have included a reference that justifies the use of a systematic mapping review (p. 5, lines 143-146).

Although one of the inclusion criteria is " Articles not published in English " it would be useful to include Spanish written articles since the reviewers are from Spain

REPLY: Both English and Spanish articles were included. 100% of the final articles included were written in English. Those that were written in another language or whose English version could not be retrieved for analysis were excluded.

Modifications were made to the text (p. 6, line 193).

Authors state on page 6 that “Two researchers independently reviewed and filtered all 3296 articles, reading and analysing the titles, abstract and keywords and following the inclusion and exclusion criteria. When the two researchers were unable to agree, a third researcher independently resolved the conflict.”
Nevertheless authors state on page 7 that “Data extraction was carried out by two researchers (DFP and ATG). Any discrepancies were resolved by two independent researchers (JML and LR).”

Are one or two researchers resolving the conflicts?

REPLY: Typing error. Conflicts were resolved by two independent reviewers.

Modifications were made to the text (p.7, line 217).

Results
Authors state on page 10 that “Of these journals, 26.6% belong to the category of neurosciences (24 articles), 21.9% to the category of experimental psychology (49 articles) and 12.5% can be considered multidisciplinary psychology journals (13 articles). Other categories include social psychology (9.4%), behavioural sciences (7.8%), clinical psychology (6.2%), developmental psychology (3.1%), multidisciplinary sciences (3.1%), geriatrics and gerontology (3.1%), medicine and experimental research (3.1%), psychiatry (1.6%) and computer graphics (1.6%).”
What criteria was used? How do authors choose if a journal belongs to several categories?

REPLY: The reviewer is right when saying that the criteria used is unclear. The classifications used by category of the journals that contained publications included in our article were extracted directly from Web of Sciences (Clarivate) under "Journal Information" and "JCR Category".

Discussion

Authors state on page19 that “To mitigate this, we conducted the search using three recognised databases”.
Nevertheless they have analysed four recognised databases (Scopus, Pubmed, PsycINFO and Web of Science).
Have the analysed three or four databases?

REPLY: We agree with the reviewer and we have corrected this error. The final articles were extracted from 4 recognised databases (Scopus, Pubmed (MEDLINE), PsycINFO and Web of Sciences).

Modifications were made to the text (p.20, line 651).

Bibliography
Please include the Journal name abbreviation (Adv Exp Soc Psychol) in the following reference:
Sedikides C, Wildschut T, Routledge C, Arndt J, Hepper EG, Zhou X. To nostalgize: Mixing memory with affect and desire. Advances in Experimental Social Psychology. 2015. 189–273. doi:10.1016/bs.aesp.2014.10.001

REPLY: The reviewer's suggestion is included.

Although it is Optional, It would be nice if you translate the article title (MEDLINE/PubMed practice) in your non English referece:
Ricarte JJ, Latorre JM, Ros L. Diseño y análisis del funcionamiento del Test de Memoria Autobiográfica en población española. Apunt Psicol. 2013;31:3–10.

REPLY: The changes suggested by the reviewer are made by translating the title of the article into English.

Figures
The PRISMA Diagram could be improved by indicating the reasons ( and the number) that are excluded at each step

REPLY: The reviewer is right and the reasons why 128 articles were eliminated in the full-text phase are clarified and described (p.7, lines, 213-215). The following text is added:

*“The reasons for articles eliminated at this stage (n=128) were as follows: 1) Wrong Intervention (n=37); 2) Wrong Age (n=61); 3) Wrong Population (n=13); 4) Wrong Design or No Results (n=9); 5) Wrong Language (n=8)”.*

Supplementary material
I wonder if English names for the S1 and S3 files attached as supplementary materials would be more appropriate than Spanish names.
Material suplementario S1.docx
Material suplementario S3.docx

REPLY: We agree with the reviewer and the correction has been made.

Recommendation: accept with minor changes, because despite these limitations, few previous studies have explored the autobiographical stimuli as a mood manipulation procedure.

REPLY: Thank you for your time and effort in the comments that allow us to improve our article.
