# Supplementary material for: Use of autobiographical stimuli as a mood manipulation procedure: Systematic mapping review
Source: PLoS One. 2022 Jun 27;17(6):e0269381. doi: 10.1371/journal.pone.0269381 (PMC9236260; doi:10.1371/journal.pone.0269381)
Supplement: Attachment — Submitted filename: Review_20220430.docx [file pone.0269381.s006.docx]

This is an interesting study that the authors exploring the Use of autobiographical stimuli as a mood manipulation procedure. This is a relevant and interesting article. However, I would like to suggest to the authors certain aspects to improve

Please change, Pubmed (MEDLINE) to Medline (PubMed).

Please dele the space the word “article” and the dot. See: we analysed a total of 126 published articles .

Please dele the space the word “topics” and the dot. See: Text mining techniques were used to extract the main related topics .

Keywords should place in alphabetical order.

Please add a reference to the first sentence “In recent years, there has been growing interest in studying emotions and their impact on different psychological processes and characteristics.”

Please change, PubMed (MEDLINE) to Medline (PubMed).

Please add an example of the search strategy

Any discrepancies should be resolved with a third researcher

Please add future implications
