# Supplementary material for: Use of autobiographical stimuli as a mood manipulation procedure: Systematic mapping review
Source: PLoS One. 2022 Jun 27;17(6):e0269381. doi: 10.1371/journal.pone.0269381 (PMC9236260; doi:10.1371/journal.pone.0269381)
Supplement: Attachment — Submitted filename: Response letter to reviewers Mapping 5-2.docx [file pone.0269381.s007.docx]

**Response letter to reviewers.**

**Ms. Ref. No.: PONE-D-21-39680**

Original Title: Use of autobiographical stimuli as a mood manipulation procedure: Systematic Mapping Review

PLOS ONE

Dear editor and reviewers,

Thank you once again for your time and dedication towards this article in making the following suggestions detailed below.

To facilitate the task of the reviewers and editors, the changes made will be detailed following the outline provided.

Kind regards.

**Reviewers' comments:**

This is an interesting study that the authors exploring the Use of autobiographical stimuli as a mood manipulation procedure. This is a relevant and interesting article. However, I would like to suggest to the authors certain aspects to improve

REPLY: Thank you very much for your comment and perspective on our research article.

Please change, Pubmed (MEDLINE) to Medline (PubMed).

REPLY: The changes indicated by the reviewers have been carried out by changing the order of the words.

Please dele the space the word “article” and the dot. See: we analysed a total of 126 published articles.

REPLY: The changes have been carried out by eliminating the marked word together with the period, connecting both sentences.

Please dele the space the word “topics” and the dot. See: Text mining techniques were used to extract the main related topics.

REPLY: The space, word and period have been removed from the marked sentence to modify it.

Keywords should place in alphabetical order.

REPLY: The keywords are sorted alphabetically

Please add a reference to the first sentence “In recent years, there has been growing interest in studying emotions and their impact on different psychological processes and characteristics.”

REPLY: Two references have been included in the text showing the scientific interest of other researchers on the subject.

1. Luminet O. (2022). Towards a better integration of emotional factors in autobiographical memory. *Memory*, 30(1), 49-54. doi: 10.1080/09658211.2021.1896738
2. Sheldon S, Chu S, Nitschke JP, Pruessner JC, Bartz JA. The dynamic interplay between acute psychosocial stress, emotion and autobiographical memory. *Sci Rep*. 2018;8, 8684. doi: 10.1038/s41598-018-26890-8

Please change, PubMed (MEDLINE) to Medline (PubMed).

REPLY: The changes indicated by the reviewers have been carried out by changing the order of the words.

Please add an example of the search strategy.

REPLY: The search string used in the databases Scopus (Elsevier), Medline (PubMed), PsycINFO (American Psychological Association) to obtain all the articles included in the figure was as follows: ("Autobiographical Memory") AND ("Emotional Induction" OR "Emotion Regulation" OR Emotion") in the title, abstract and keywords fields for their final result. . This string of words was used in each of them by selecting articles between the years 2000 and 2021.

Any discrepancies should be resolved with a third researcher.

REPLY: This has been modified in the text. All discrepancies that arose during article filtering were resolved by a third reviewer.

Please add future implications.

REPLY: In the final part of the discussion, we add the future implications based on the evidence shown in the resulting articles:

“Given the importance of emotions in people’s day-to-day lives and their effect on associated psychological variables, future lines of research that may arise from the evidence allow us to advance in the study of procedures based on less commonly used autobiographical stimuli, such as photographs. Additionally, we propose investigating different associations of both the type of photo or memory, together with an exhaustive examination of the variable itself, measuring the impact and its consequences on mood. In light of the methodologies analysed, this article lays the groundwork for the importance of establishing a rigorous and clearly defined protocol for better interventions based on mood induction through AM in experimental and natural settings. Furthermore, a new avenue of research would be to conduct meta-analyses that quantitatively reflect the potential of such procedures. Future studies could also be directed in terms of relating the findings of the present review to another specific field of study and context. The methodology applied in this study is well documented and could therefore be replicated on future occasions.”
